# Causal Thinking and Trauma Determine Dementia Prevention Behaviors in Indigenous and Non-Indigenous Older Adults

**DOI:** 10.1093/geroni/igaf122.4377

**Published:** 2025-12-31

**Authors:** Jose Aravena, Moises Sandoval, Javiera Provis, Carolina Herrera, Karen Wang, Cecilia Albala, Becca Levy

**Affiliations:** Yale University, New Haven, Connecticut, United States; INTA, Universidad de Chile, Santiago, Region Metropolitana, Chile; ComunidadMujer, Santiago, Region Metropolitana, Chile; Hospital Hernan Henriquez Aravena, Temuco, Araucania, Chile; Yale University, New Haven, Connecticut, United States; INTA, University of Chile, Santiago, Region Metropolitana, Chile; Yale School of Public Health, New Haven, Connecticut, United States

## Abstract

Indigenous groups in high-income countries experience a higher risk of dementia than non-Indigenous populations. Internal causal thinking (attributing chronic diseases to factors within the individual) has been linked to healthier behaviors, but its role in cognitive health, and how trauma perceptions shape preventive behaviors, remains unclear. We investigated whether causal thinking about cognitive problems and the identification of traumatic experiences as a cause influence engagement in dementia prevention behaviors among Indigenous and non-Indigenous older adults in Chile. In a sequential mixed-method study, Mapuche (n = 167) and non-Indigenous (n = 245) adults aged ≥60 years completed open-ended questions to classify internal versus external causal thinking toward memory problems and Alzheimer’s disease (AD), and whether trauma was perceived as a cause. Dementia prevention behaviors were measured by engagement in healthy lifestyles and lifestyle-related dementia risk factors. Fully adjusted multivariate regressions showed that internal causal thinking toward memory problems and AD was associated with better prevention behaviors in the total sample (memory loss: B = −0.028, SE = 0.011, p = 0.015; AD: B = −0.043, SE = 0.014, p = 0.002). Among Mapuche participants, this association was present only for memory problems (B = −0.046, SE = 0.019, p = 0.017), while among non-Indigenous participants it was present only for AD (B = −0.052, SE = 0.018, p = 0.004). Notably, Mapuche participants were more likely to identify trauma as a cause of cognitive problems, and this was linked to more dementia risk factors (B = 0.536, SE = 0.222, p = 0.017). These findings suggest that trauma-related causal beliefs may be an important, culturally specific barrier to dementia prevention, underscoring the need to address trauma narratives alongside health education in prevention strategies

